# Atypical Cardiac Tamponade Manifesting as Left Ventricular Diastolic Collapse: A Case Report

**DOI:** 10.7759/cureus.8045

**Published:** 2020-05-10

**Authors:** Shweta Paulraj, Vijay Raj, Prashanth Ashok Kumar, Robert Voelker, Harold Smulyan

**Affiliations:** 1 Internal Medicine, State University of New York Upstate Medical University, Syracuse, USA; 2 Cardiology, State University of New York Upstate Medical University, Syracuse, USA

**Keywords:** pericardial effusion, echocardiogram, valve replacement, tamponade, cardiac surgery, diastolic collapse, chamber collapse, paradoxical septal motion, hemopericardium

## Abstract

Cardiac tamponade is a medical emergency, the diagnosis of which is predominantly clinical with supportive echocardiographic findings. Echocardiographic findings highly suggestive of cardiac tamponade include chamber collapse, inferior vena cava (IVC) plethora, and respiratory volume/flow variations. The right-sided cardiac chambers are a low-pressure system and are the first to show signs of collapse with high specificity for tamponade. We report the case of a 35-year-old woman who demonstrated left ventricular (LV) diastolic collapse on echocardiogram following a tricuspid valve replacement. Although left-sided chamber collapse with tamponade has been reported with localized pericardial effusions postoperatively, our patient had a large circumferential pericardial effusion. Selective chamber compression can be a presenting sign of postoperative tamponade after cardiac surgery. Our case highlights the importance of recognizing atypical forms of cardiac tamponade to help in early identification and emergent management in such patients.

## Introduction

Cardiac tamponade is a medical emergency that requires timely recognition and treatment [1]. It is predominantly a clinical diagnosis with Beck’s triad, comprising hypotension, distended neck veins, and muffled heart sounds, present only in a minority of patients [2]. Echocardiography aids in the diagnosis of cardiac tamponade by confirming the presence of fluid and using chamber collapse to assess its hemodynamic impact [3]. Early diastolic collapse of the right-sided chambers is a frequent and specific finding for cardiac tamponade, but the collapse of the left ventricle (LV) is rare [4]. We report the case of a patient with cardiac tamponade manifesting as LV diastolic chamber compression on echocardiography.

## Case presentation

A 35-year-old African-American woman with sickle cell disease presented to the hospital with severe, progressive dyspnea and a sickle cell crisis. She had a history of severe tricuspid regurgitation and had been previously treated for protein C deficiency, recurrent deep vein thrombosis, and pulmonary emboli with an inferior vena cava (IVC) filter and chronic warfarin therapy. On examination, she had 2+ pitting pedal edema, jugular venous distension, a grade 2/6 systolic murmur over the tricuspid area, tender hepatomegaly, and diffuse abdominal distension. Echocardiogram revealed severe tricuspid regurgitation, a markedly dilated right atrium, dilated tricuspid annulus, and a mildly dilated but normally functioning right ventricle (RV). Doppler findings suggested moderate pulmonary hypertension with a pulmonary artery systolic pressure of 55 mmHg. These findings led the doctors to conduct a bioprosthetic tricuspid valve replacement surgery.

**Figure 1:**
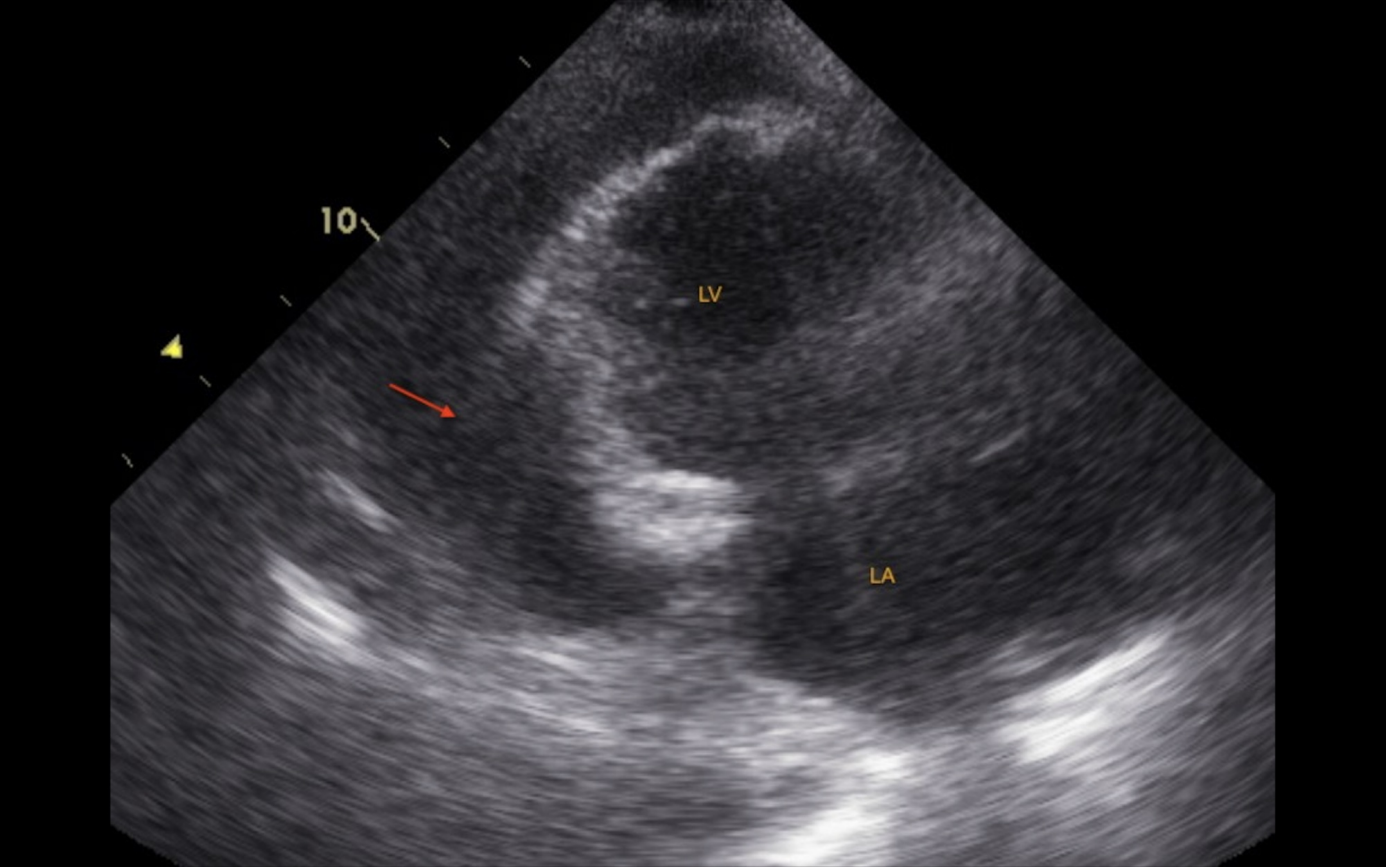
Transthoracic echocardiogram: apical two-chamber view The image shows large circumferential pericardial effusion, predominantly posterior in a location with fibrinous strands (arrow) LA: left atrium; LV: left ventricle

Postoperatively, she was hemodynamically stable, had crackles on lung auscultation and hypoxic respiratory failure that required 2-3 L/minute of oxygen via a nasal cannula, intermittent bilevel positive airway pressure ventilation, and diuresis. Twenty days after surgery, purulent secretion was noted on the sternotomy site. A CT of the thorax showed a large pericardial effusion, possibly a hemopericardium, and bilateral pleural effusions. Warfarin was discontinued. The echocardiogram showed a large circumferential pericardial effusion, predominantly posterior in a location with fibrinous strands (Figure 1, Video 1).

**Video 1:** Transthoracic echocardiogram: apical four-chamber view showing LV collapse The video shows large circumferential pericardial effusion with diastolic collapse of the LV LA: left atrium; LV: left ventricle; RA: right atrium; RV: right ventricle

**Figure 2:**
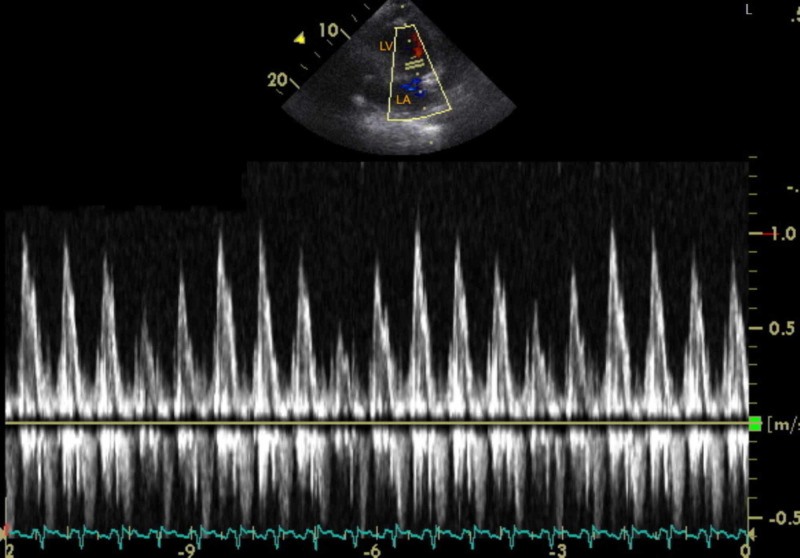
Transthoracic echocardiogram with pulse wave doppler The image shows mitral inflow velocity on pulse wave doppler showing about 40% respiratory-driven variation LA: left atrium; LV: left ventricle

**Figure 3:**
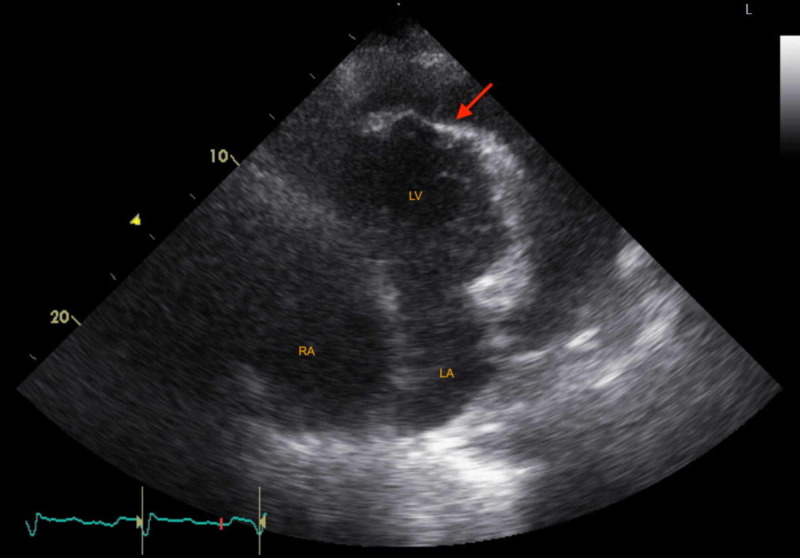
Transthoracic echocardiogram with LV collapse Apical-four chamber view showing large pericardial effusion with diastolic LV apical collapse (arrow) LA: left atrium; LV: left ventricle; RA: right atrium

There was paradoxical interventricular septal motion with LV septal bounce, excessive mitral inflow respiratory driven variation of ~40% (Figure 2), and LV apical diastolic compression (Figure 3, Video 1). The prosthetic tricuspid valve functioned normally.

**Figure 4:**
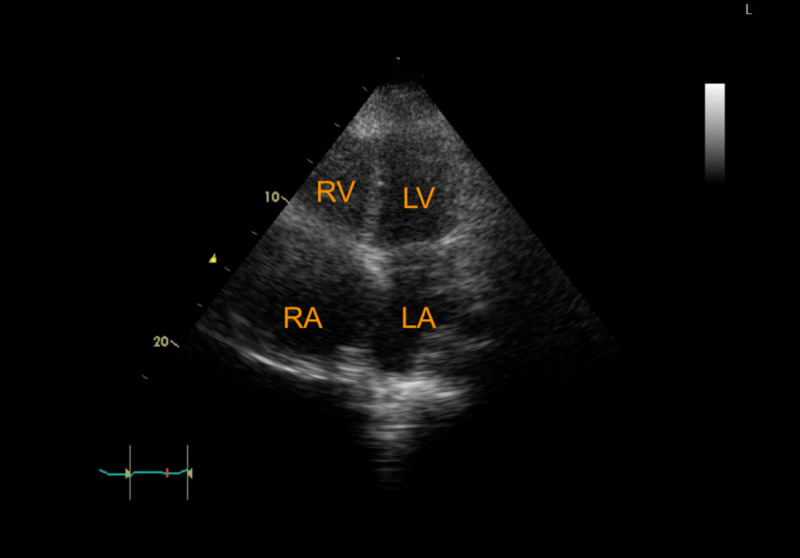
Postoperative echocardiogram with no pericardial effusion Apical four-chamber view showing RA, RV, LA, and LV LA: left atrium; LV: left ventricle; RA: right atrium; RV: right ventricle

She underwent a subxiphoid pericardial window and placement of a drainage catheter, which led to the removal of 500 cubic centimeters of blood followed by a gradual improvement in her symptoms. A follow-up echocardiogram two months after discharge showed no pericardial effusion (Figure 4).

## Discussion

Pericardial effusions can be secondary to infections, inflammatory diseases, infarction, malignancy, valve replacements, and thoracotomy [5]. Effusions with cardiac tamponade are more common following valve surgery than after coronary artery bypass graft surgery, with a reported incidence of 0.6-4.3% [6]. Various causative mechanisms have been postulated, the chief one being anticoagulation. Postoperative cardiac tamponade is reported to be more common in females, and any amount of pericardial effusion on the first postoperative transthoracic echocardiogram has been found to be predictive of cardiac tamponade. Despite prolonged hospital stays and high readmission rates in patients with postoperative cardiac tamponade, its course is usually benign if intervened in a timely manner [7].

Significant echocardiographic findings in tamponade include chamber collapse, commonly of the right-sided chambers, IVC plethora, and excessive respiratory variation in cardiac inflow volume. The intrapericardial pressure exceeds the chamber pressure in patients with tamponade, and the low-pressure right-sided chambers usually show the first signs of collapse [8]. The LV is thicker, less compliant, and encloses a high-pressure system that tends to resist collapse [3]. Interventricular septal bounce on the echocardiogram is highly suggestive of cardiac tamponade with increased ventricular interdependence [9].

LV diastolic collapse has been described as a sign of cardiac tamponade following cardiac surgery [10]. These effusions can be located posteriorly in 49% of the patients but also may be compartmentalized, causing a diastolic collapse of the LV rather than the RV [6]. Loculated effusions due to adhesions can cause the typical hemodynamic abnormalities in the compressed chambers only and therefore posterior pericardial effusions can lead to isolated LV cardiac tamponade [11]. Another scenario that could explain the left side chamber collapse is pulmonary artery hypertension where increased right heart pressures may exceed pericardial pressures and prevent right-sided collapse. Left-sided filling pressures may then be lower than pericardial pressures in such patients, producing collapse of the left atrium [3]. Our patient had evidence of moderate pulmonary hypertension in her initial echocardiogram.

Echocardiographic detection of chamber collapse, increased respiratory-driven valvular flow variation, and reduced hepatic venous flow all suggest imminent cardiovascular collapse and require urgent treatment. Echocardiographic guided pericardiocentesis and the removal of even a small amount of pericardial fluid can result in a substantial drop in the intrapericardial pressure and improvement in symptoms [8]. Catheter drainage of the pericardial fluid and surgical drainage via a pericardial window are other options in patients with hemopericardium (as in our patient), purulent pericarditis, or neoplastic effusions with a high risk for recurrence [12,13].

## Conclusions

Atypical forms of cardiac tamponade with varied clinical presentations may be seen in patients after cardiac surgery, often with selective chamber compression. An awareness of these uncommon presentations of cardiac tamponade is essential for prompt and appropriate emergency care of these patients.
